# Molecular Analysis of a Recurrent Sarcoma Identifies a Mutation in FAF1

**DOI:** 10.1155/2015/839182

**Published:** 2015-03-10

**Authors:** Georg F. Weber

**Affiliations:** College of Pharmacy, University of Cincinnati, 3225 Eden Avenue, Cincinnati, OH 45267-0004, USA

## Abstract

A patient presented with a recurrent sarcoma (diagnosed as leiomyosarcoma) 12 years after the removal of an initial cancer (diagnosed as extracompartmental osteosarcoma) distally on the same limb. Following surgery, the sarcoma and unaffected muscle and bone were subjected to measurements of DNA exome sequence, RNA and protein expression, and transcription factor binding. The investigation provided corroboration of the diagnosis leiomyosarcoma, as the major upregulations in this tumor comprise muscle-specific gene products and calcium-regulating molecules (calcium is an important second messenger in smooth muscle cells). A likely culprit for the disease is the point mutation S181G in FAF1, which may cause a loss of apoptotic function consecutive to transforming DNA damage. The RNA levels of genes for drug transport and metabolism were extensively skewed in the tumor tissue as compared to muscle and bone. The results suggest that the tumor represents a recurrence of a dormant metastasis from an originally misdiagnosed neoplasm. A loss of FAF1 function could cause constitutive WNT pathway activity (consistent with the downstream inductions of IGF2BP1 and E2F1 in this cancer). While the study has informed on drug transport and drug metabolism pharmacogenetics, it has fallen short of identifying a suitable target for molecular therapy.

## 1. Introduction

Sarcomas are cancers of mesenchymal origin [1] that comprise about 1% of adult malignancies. Leiomyosarcomas are derived from smooth muscle cells. At most primary sites, other than the uterus or gastrointestinal tract, leiomyosarcomas are likely to originate from the tunica media of blood vessels. However, it has been postulated that primary leiomyosarcoma of the bone might also develop through advanced myogenic metaplasia of a sarcoma originating from fibroblastic tissue [2]. The disease typically occurs in the 5th to 6th decades of life, with women being affected more than men (2 : 1). This gender distribution may reflect the proliferation of smooth muscle that can occur in response to estrogen [3].

Because sarcomas tend to respond poorly to standard chemotherapy, they have no good treatment options beside total excision with wide margins. However, the gradual replacement of the highly toxic conventional cancer chemotherapy (comprising nonspecific antiproliferative agents) with molecularly targeted drugs, which was initiated with the market entry of neutralizing antibodies and small molecule kinase inhibitors in 1997 (Rituxan) and 2001 (Gleevec), respectively, has opened the possibility to tailor drug treatment to particular tumors. Yet, this transition has also necessitated the molecular characterization of the lesions that are causative for the transformation of healthy cells to cancerous cells because drugs need to be matched with the underlying carcinogenic defect to be effective. Here, we take a sarcoma through a comprehensive molecular analysis that applies multiple screening techniques, with the goal to identify the disease-causing defects as well as potential drug targets.

## 2. Materials and Methods

### 2.1. Patient and Tissues

A 52-year-old female patient underwent surgery for a recurrent sarcoma. Samples of skeletal muscle, bone, and tumor were obtained postsurgery.

### 2.2. DNA Exome Sequencing

1 *μ*g of dsDNA determined by Invitrogen Qubit high sensitivity spectrofluorometric measurement was sheared by sonication to an average size of 300 bp on a Diagenode Bioruptor. Automated library construction was performed on an IntegenX Apollo324 which size-selects fragments by double-SPRI binding with different concentrations of PEG for a high cut and a low cut. Each library can be fitted with one of 48 adapters, each containing a different 6-base molecular barcode for high level multiplexing. After 12 cycles of PCR amplification, 1 *μ*g of genomic library was recovered for exome enrichment using the NimbleGen EZ Exome V2 kit. Enriched libraries were sequenced on an Illumina HiSeq2000, generating around 32 million high quality paired end reads of 100 base each or 6.4 GB of usable sequence per sample. The analysis methods utilize the Broad Institute's Genome Analysis Toolkit (GATK) and follow a pipeline previously described [4], along with published modifications (http://www.broadinstitute.org/gsa/wiki/index.php/The_Genome_Analysis_Toolkit). The analysis comprises aligning the reads that pass Illumina Chastity Filter with the Burrows-Wheeler Aligner (BWA) [5]. For each sample, Picard's MarkDuplicates are used to flag reads that appear to be artifacts of PCR bias. All reads that overlap known or putative indels are realigned. All base quality scores are recalibrated to the empirical error rate derived from nonpolymorphic sites. The GATK's Unified Genotyper module is used to call variant sites (both single nucleotide and small indel) in all samples simultaneously. Finally, the SNV calls are filtered using the variant quality score recalibration method [4]. Indel calls were filtered with a set of hard filters, as there are not enough indels in an exome to use the Gaussian method.

### 2.3. RNAseq

Tissue samples were homogenized in RNazol RT (MRC) with a manual homogenizer and stored on ice until extraction. The RNA isolation was performed according to the manufacturer's instructions.

The Ovation RNA-Seq FFPE system (NuGen) was used to initiate amplification at both 3′ end as well as randomly throughout the transcriptome in the sample. 100 ng of total RNA with RIN < 5.0 was converted into a library of template molecules suitable for subsequent cluster generation and sequencing by Illumina HiSeq. Total RNA was reverse transcribed and converted to double stranded cDNA with a unique DNA/RNA heteroduplex at one end. NuGEN's Ribo-SPIA technology was used for isothermal amplification resulting in the rapid generation of cDNA with a sequence complementary to the original mRNA. The cDNA was then double stranded and fragmented to 200 bp using Covaris S2, and a sequencing library was generated using Illumina's TruSeq DNA Sample Prep Kit V2 according to standard protocols. The cDNA library was enriched by a limited number of 10 PCR cycles, validated using an Agilent 2100 Bioanalyzer, and quantitated using the Quant-iT dsDNA HS Kit (Invitrogen). Two individually indexed cDNA libraries were pooled and sequenced on Illumina HiSeq to get a minimum of 90 million reads. Libraries were clustered onto a flow cell using Illumina's TruSeq SR Cluster Kit v2.5 and sequenced 50 cycles using TruSeq SBS Kit-HS on HiSeq. The obtained sequence reads were aligned to the genome by using the standard Illumina sequence analysis pipeline.

### 2.4. Protein-DNA Array

Tissues were ground between frosted glass slides and then incubated with Collagenase and Dispase in cell culture medium at 37°C for 45 minutes to release individual cells. These cells were collected after passing the samples through a strainer and centrifugation. Nuclear extracts and cytosol were prepared using a kit from Active Motif. After protein determination, DNA binding of the nuclear extracts was assessed with the Combo protein-DNA array (Panomics). Signal intensity was measured with the software MetaMorph.

### 2.5. Western Blotting

For the analysis of individual proteins, tissues were homogenized in RIPA buffer (50 mM Tris-HCl pH 7.5, 150 mM NaCl, 1% NP-40, 0.1% sodium dodecyl sulfate) using a handheld, battery-operated homogenizer. 10 *μ*g lysates were loaded per lane and electrophoresed on 10% SDS-polyacrylamide minigels with reducing, denaturing sample buffer. The separated proteins were transferred to PVDF membranes and probed with antibody O-17 (IBC) to the C-terminus of osteopontin and anti-HCAM to the cytoplasmic domain of CD44 (Santa Cruz) and to STAT3 and phospho-STAT3 (Cell Signaling Technology). Antitubulin serves as a loading control.

### 2.6. 2D Gel Electrophoresis and Mass Spectrometry

The tumor and muscle samples were diluted to 4 and 1 mg/mL in 1 : 1 diluted SDS Boiling Buffer : Urea Sample Buffer before loading (the bone sample was ethanol precipitated and redissolved to 4 and 1 mg/mL in 1 : 1 diluted SDS Boiling Buffer : Urea Sample Buffer). Two-dimensional electrophoresis was performed according to the carrier ampholine method of isoelectric focusing [9, 10] by Kendrick Labs, Inc. Isoelectric focusing was carried out in glass tubes of inner diameter 2.3 mm using 2% pH 4–8 Servalytes (Serva, Germany) for 9600 volt-hours. 1 *μ*g (Coomassie stain) or 50 ng (silver stain) of an IEF internal standard, tropomyosin, was added to each sample. This protein migrates as a doublet with lower polypeptide spot of MW 33,000 and pI 5.2; its position is marked by an arrow on the stained gels. The enclosed tube gel pH gradient plot for this set of ampholines was determined with a surface pH electrode.

After equilibration for 10 min in buffer “O” (10% glycerol, 50 mm dithiothreitol, 2.3% SDS, and 0.0625 M Tris, pH 6.8), each tube gel was sealed to the top of a stacking gel that was on top of a 10% acrylamide slab gels (0.75 mm thick). SDS slab gel electrophoresis was carried out for about 4 hours at 15 mA/gel. The following proteins (Sigma Chemical Co.) were used as molecular weight standards: myosin (220,000), phosphorylase A (94,000), catalase (60,000), actin (43,000), carbonic anhydrase (29,000), and lysozyme (14,000). These standards appear along the basic edge of the Coomassie blue R-250 stained or silver-stained [11] 10% acrylamide slab gel. The gels were dried between sheets of cellophane paper with the acid edge to the left. Each of the gels was overlaid with a transparent sheet for labeling polypeptide spot differences without marking the original gel (Kendrick Labs).

### 2.7. Polymorphism Analysis

We obtained 22 formalin-fixed, paraffin-embedded leiomyosarcoma specimens (stroma and paraffin had been removed from unstained slides under microscopic examination) through the Department of Pathology, University of Cincinnati. DNA was extracted with the AllPrep DNA/RNA FFPE kit (Qiagen). We purchased 7 frozen leiomyosarcoma tissues from Creative Bioarray and extracted DNA with the AllPrep DNA/RNA Mini Kit (Qiagen). One blood sample from a leiomyosarcoma patient was received from the University of Cincinnati Tissue Bank. Polymorphisms in FAF1 were analyzed in the DNA using a custom TaqMan assay with the probe ACACCAGATTTGCCACCACCTTCATCATCT [A/G] GTCATGCTGGGTAAGTTGTTTATATTTCCTG. A TaqMan assay with an existing probe for position −443 in the osteopontin promoter served as a reference assay. 34 breast cancer DNA samples served as nonsarcoma control. The assay was performed by the CCHMC DNA core.

## 3. Results

### 3.1. Patient History

**Figure 1:**
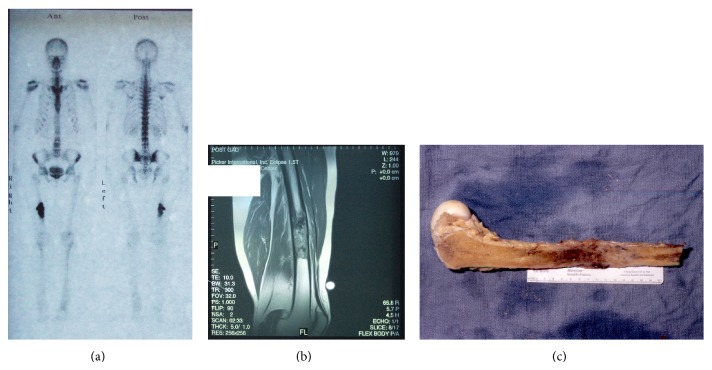
Osteosarcoma. (a) Whole body scan shows abnormally intense uptake of the radionuclide (Tc) within the middiaphysis of the right femur. No increased radionuclide uptake is seen anywhere else in the bony skeleton. (b) MRI scan displays an extensively abnormal signal in the diaphysis of the right femur. It is surrounded by soft tissue involvement. (c) Bone lesion displayed postoperatively.

In 1998, the patient was diagnosed with high grade, stage IIb osteogenic sarcoma of the right femur, which was extracompartmental. Upon resection the lesion had a size of 9.5 × 3 × 4 cm (Figure 1). The tumor was assessed as stage T2NxMx, grade III, characterized as hypercellular; it showed marked cytologic atypia and a high mitotic rate. Histologic features included anaplasia, pleomorphism, numerous abnormal mitoses, numerous giant cells, and osteoid production with focal calcifications.

In 2012, a CT scan, done because of hip pain, revealed a 4.3 × 3.4 × 3.1 cm lytic mass in the superior right acetabulum, grossly stable in size and configuration. There was diffuse osteopenia involving the right femoral head and neck with diffuse atrophy of the right pelvic girdle musculature. Periostitis and cortical interruption were associated with this lesion.

The postsurgical pathology report identified the 5.7 × 5.5 × 4.9 cm mass as a high grade leiomyosarcoma, stage pT2bpNx. Whereas a bone scan revealed intense activity in the left seventh rib, a follow-up chest CT provided no indication of pulmonary masses, mediastinal or hilar lymphadenopathy, enlargement of axillary nodes, or pleural effusion, implying stage M0. The mitotic rate was 70%, with 60% necrosis. The tumor caused extensive bone destruction and involvement of adjacent tissue. Histologically, the tumor cells stained positively for smooth muscle actin. They were also positive for CD68 and displayed diffuse positive staining for vimentin but were negative for CD117, pancreatin, S-100, and CD34.

Seven months after the surgery, the patient received a PET-MRI scan for pain, which revealed six metastatic lesions, including both lungs and multiple ribs. She was put on three 21-day cycles of Gemzar (days 1 and 8), Taxotere (day 8), and Neulasta (beginning on day 9) but was unable to continue past the first cycle due to hospitalizations for continued and problematic wound infections at the surgical lung biopsy sites.

### 3.2. DNA Exome Sequence

**Figure 2:**
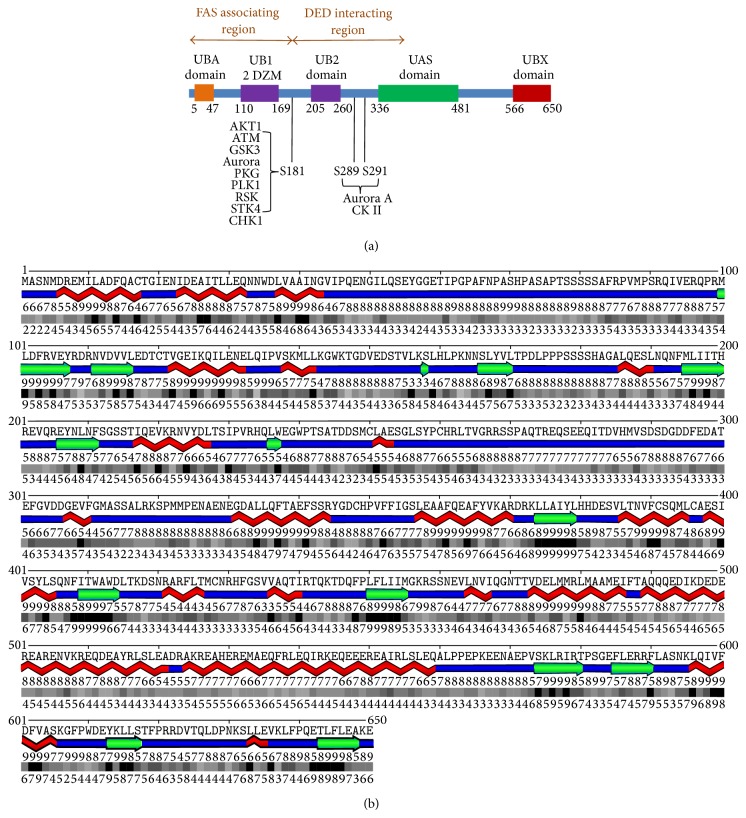
FAF1 structure. (a) A schematic of functional domains in FAF1 with annotations (adapted from [6–8]). There are two ubiquitin-like domains flanking the S181 site. The PDB structure 2DZM identifies the N-terminal one, while the C-terminal prediction is by sequence similarity. Whereas the mutation S181G is not expected to disrupt the protein structure per se, this amino acid has a high score as a possible phosphorylation site for a number of kinases involved in DNA damage repair. (b) Secondary structure prediction of FAF1. The residues around S181 appear unstructured.

Exome sequencing of the genomic DNAs for tumor, muscle, and bone identified 65546 potential sequence variants. Filtering yielded 46 likely somatic mutations in the tumor (Table 1), of which 7 (affecting EIF4A1, EPHA3, FAF1, IPO8, KIAA1377, LIMCH1, and NIPBL) were confirmed in the RNASeq results. FAF1 associates with FAS and enhances apoptosis mediated through this receptor [12]. The point mutation S181G (Figure 2) could cause a loss of function in FAF1 and lead to transformation via antiapoptosis.

### 3.3. RNA Analysis

Expectedly, the gene expression patterns, according to RNASeq, were very different among tumor, muscle, and bone. The cancer contained several gene products that were overexpressed compared to both muscle and bone (Tables 2(a) and 2(b)). Among the top 30 changes in the tumor/muscle and tumor/bone comparisons, 13 were identical (Table 2(c)). It is implied that these gene products are quite unique for the tumor and likely contribute to its pathogenesis. This notion is supported by the upregulation of the smooth muscle gene Ano4, which corroborates the leiomyosarcomatous nature of the cancer. When limiting the analysis to genes expressed at least at the level of 1 unit, the 17 genes overexpressed in the tumor/muscle and tumor/bone comparisons contain several extracellular matrix proteins, implying an active remodeling of the tumor microenvironment (Table 2(d)). By contrast, none of the underexpressed gene products in the tumor/muscle comparison matched the tumor/bone comparison (not shown).

Of note, the RNA level of IGF2BP1 (IMP-1, CRD-BP, and ZBP-1) is highly upregulated in the tumor compared to muscle as well as bone. IGF2BP1 is a RNA-binding factor that affects mRNA nuclear export, localization, stability, and translation. It regulates mRNA stability during the integrated cellular stress response in stress granules. IGF2BP1 is a transcriptional target of the WNT pathway, which is negatively regulated by intact FAF1 and may be unregulated by FAF1^S181G^. The IGF system has been linked to sarcoma pathogenesis [13] and may play a role in this specific cancer. Other IGF family members with increased RNA message levels in this tumor (compared to muscle and bone) include IGFBPL1 (5-6-log_2_-fold), IGFL3 (6-7-log_2_-fold), and IGF2BP3 (2-6-log_2_-fold).

The identified point mutation in FAF1 may be pathogenetic for this cancer. FAF1 is a regulator of NF-*κ*B activation. It directly binds to RelA (P65), retaining it in the cytoplasm. It can also interact with IKK*β*, thus allowing for the I*κ*B-mediated degradation of the transcription factors P65 and P50 [6]. Consistently, the expression of regulators of the NF-*κ*B activation pathway is skewed in the tumor compared to muscle or bone (Table 2(e)).

### 3.4. Transcription Factor Binding

**Figure 3:**
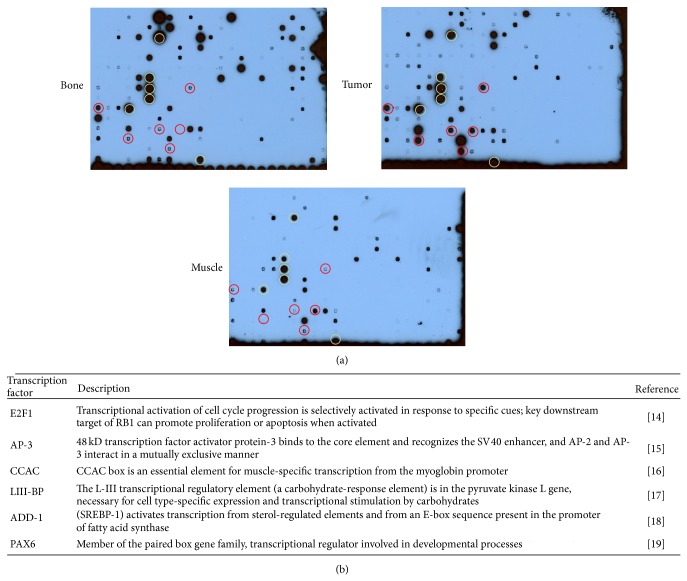
Transcription factor activation. Nuclear extracts from tumor, muscle, and bone were tested for DNA binding activity on a protein-DNA array. (a) Transcription factor binding that was high in all 3 tissues and is therefore considered constitutively active is circled in yellow (left to right, top to bottom: AhR/Amt, GATA1, GATA2, GATA1/2, HIF1, and HOXD8/9/10). Transcription factors that show high binding in the cancer, but not in muscle or bone, are circled in red (left to right, top to bottom: E2F1, AP3, LIII-BP, PAX6, ADD-1, and CCAC). (b) The functions of transcription factors that display high DNA binding in the cancer, but not in muscle or bone, are described.

Protein/DNA arrays measure the binding activity of transcription factors. They comprise three basic steps. A set of biotin-labeled DNA binding oligonucleotides are preincubated with a nuclear extract of interest. The protein/DNA complexes are separated from the free probes. The probes in the complexes are then extracted and hybridized to prespotted membranes followed by HRP-based chemiluminescence detection. We made nuclear extracts from tumor, bone, and muscle and tested them for DNA binding activity. Binding that was induced in cancer, but not in the normal tissues, was displayed by the transcription factors E2F1, AP3, LIII-BP, PAX6, ADD-1, and CCAC [14–19]. The CCAC binding activity is consistent with a muscle-derived tumor. E2F1 may associate with the WNT pathway-induced transcription factor LEF1, resulting in transcriptional derepression of E2F1 [20]. Likely constitutive transcription factors that are active in all 3 tissues comprise AhR/Amt, GATA1, GATA2, GATA1/2, HIF1, and HOXD8/9/10 (Figure 3).

### 3.5. Protein Analysis

**Figure 4:**
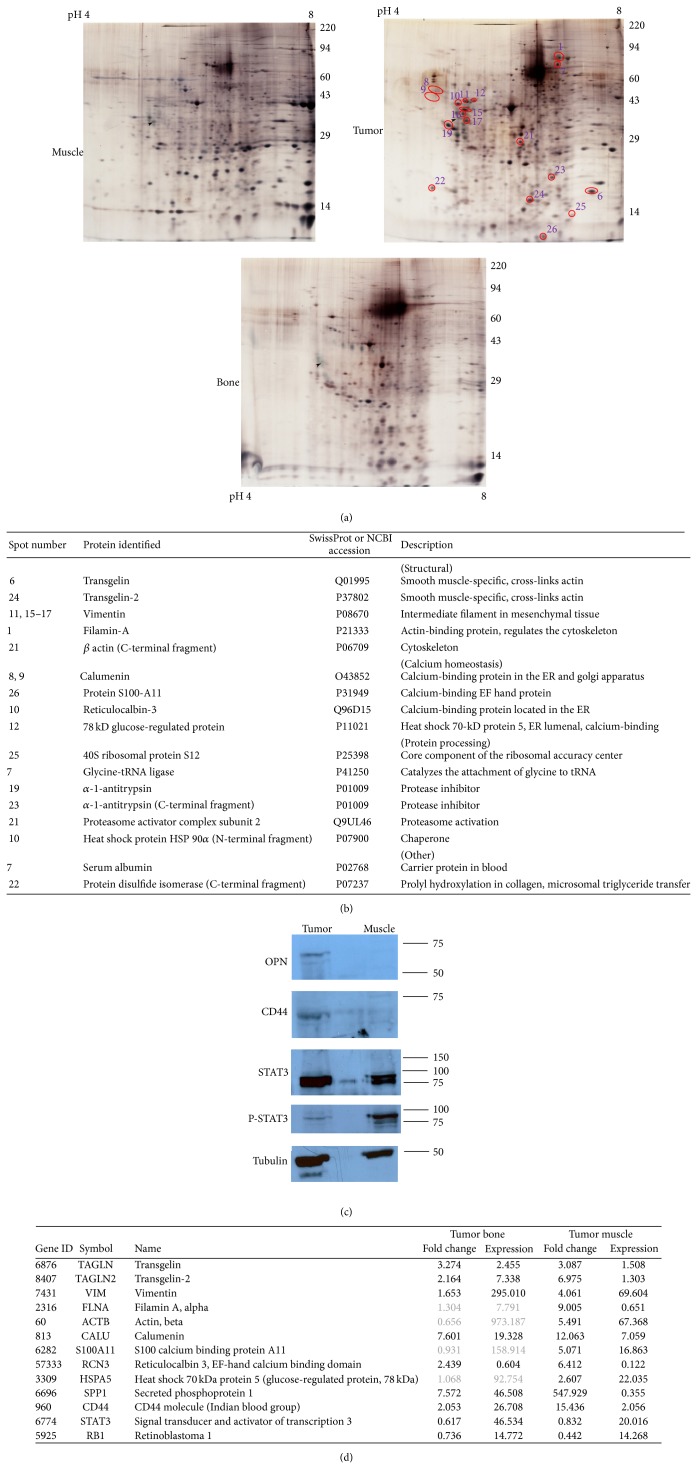
Protein overexpression. (a) 2D protein gel electrophoresis of lysates from muscle (upper left), tumor (upper right), and bone (bottom) in RIPA buffer. Red circles indicate the spots that were identified as overexpressed and were further analyzed by mass spectrometry. (b) Proteins identified in 2D gel electrophoresis as overexpressed in the tumor in comparison to muscle and bone were analyzed for their identity by mass spectrometry. The left column indicates the spot number corresponding to the 2D gel. The next column contains the protein name, followed by the accession number and a description of the protein function. The protein functions are grouped into structural, calcium homeostasis, and various others. (c) Western blot. 10 *μ*g lysates of tumor, muscle, and bone in RIPA buffer were loaded per lane and electrophoresed on 10% SDS-polyacrylamide minigels with reducing, denaturing sample buffer. After transfer to PVDF membranes, they were probed for markers of cancer progression, including osteopontin, CD44, STAT3, and phospho-STAT3. Antitubulin served as a loading control. (d) RNA levels corresponding to the proteins found to be affected in the sarcoma. With four exceptions in the tumor-bone comparison (gray font), upregulated proteins are associated with increased RNA levels. STAT3 is not increased on the protein or RNA level. The reduced level of RB1 expression is consistent with the elevated DNA binding activity of E2F1.

2D gel electrophoresis of the RIPA lysates from tumor, muscle, and bone showed very divergent patterns (Figure 4(a)). Two experienced analysts compared the protein pattern from the tumor with the protein pattern from either bone or muscle. Polypeptide spots that were unique to the gels from the tumor were outlined (spots unique to or relatively darker in the bone or muscle were not indicated). The labeled proteins were extracted for identification with mass spectrometry. This yielded several structural proteins, which may reflect modification of the cellular architecture under rapid growth. Transgelin-1 and transgelin-2 were abundant and corroborated the identity of the tumor as a leiomyosarcoma. Four calcium-binding proteins were highly expressed in the cancer. In addition, regulators of protein synthesis (40S ribosomal protein S12, glycine-tRNA ligase), protein modification (N-terminal fragment of heat shock protein HSP 90*α*, C-terminal fragment of protein disulfide isomerase), and protein degradation (*α*1-antitrypsin, proteasome activator complex subunit 2) were identified (Figure 4(b)). Of interest may be the C-terminal fragment of protein disulfide isomerase, which not only hydroxylates prolines in preprocollagen but also contributes to microsomal triglyceride transfer. It could be reflective of a skewed tumor metabolism. The protein analysis was corroborated by the mRNA levels (Figure 4(d)).

Cancer markers were tested according to Western blot (Figure 4(c)). The tumor, but not normal muscle, expressed the metastasis protein osteopontin and a single small form (<75 kD) of CD44 that is likely the not alternatively spliced, standard form. Unexpectedly, while both tumor and muscle expressed comparably abundant amounts of STAT3, phosphorylation (reflective of activation) was present in the muscle but not in the tumor. The STAT3 pathway is associated with progression in several human cancers, and this is often reflected in STAT3 constitutive phosphorylation. The lack of phosphorylation in this case suggests that the leiomyosarcoma may not depend on the STAT3 pathway.

### 3.6. Pharmacogenetic Evaluation

Predicting the sensitivity to anticancer drugs is a main goal of molecular analysis. For this, the over- or underexpression of genes for drug transport and metabolism is of key importance. Analysis of the RNASeq data for these groups of gene products identified a surprisingly large number of deregulations compared to muscle or bone (Tables 3(a) and 3(b)). Those alterations may affect choices for drug treatment. For example, the high levels of glutathione S-transferase may render carmustine, thioTEPA, cisplatin, chlorambucil, melphalan, nitrogen mustard, phosphoramide mustard, acrolein, or steroids ineffective. The overexpression of* N*-acetyltransferase may compromise 5-fluorouracil or taxol. The modest upregulation of only two export transporters (ABC-transporters), and specifically the lack of ABCB1 overexpression, is favorable for avoiding drug resistance.

### 3.7. Population Analysis

The above-described results indicated that a FAF1 mutation, which replaces serine in position 181, thus preventing FAF1 phosphorylation and activation, may be a driver for leiomyosarcomagenesis. A custom TaqMan assay confirmed the presence of the somatic mutation in the patient. To assess whether this single nucleotide replacement is common in this type of cancer, we analyzed DNA from 29 leiomyosarcomas and 1 blood sample from a leiomyosarcoma patient. For comparison to nonsarcomatous tumors, 34 breast cancers served as a reference. None of them displayed a mutation in the same locus. By contrast, there was a distribution across all leiomyosarcomas in the osteopontin promoter position −443 (used as a reference), with 12 CC, 10 TC, and 9 TT.

## 4. Discussion

**Figure 5:**
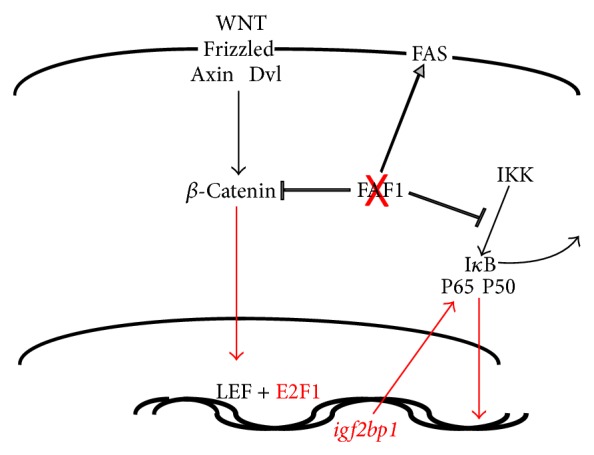
Possible transforming pathway. The point mutation in FAF1 is believed to cause a loss of function (crossed out in red). This may lead to an overactivity of the WNT signaling pathway. Consistently, E2F1 (activated by LEF1) and* igf2bp1* (a transcriptional target of the WNT pathway) have been identified to be upregulated in the molecular analysis. In contrast to the negative regulator FAF1, IGF2BP1 is a positive regulator of NF-*κ*B activity. The overactivity of the NF-*κ*B pathway and the reduced efficacy of FAS signaling can be transforming.

The FAF1 mutation identified as the likely cause for the cancer under study gives room for an explanation of the sarcomatous transformation (Figure 5). DNA damage to mesenchymal cells occurs persistently in an oxidizing environment at 37°C. These insults are rarely transforming, and such an occurrence would trigger the initiation of programmed cell death in apoptosis-competent cells. Intact FAF1 associates with FAS and enhances apoptosis mediated through this receptor [12]. A loss of function in FAF1 could lead to transformation via antiapoptosis. Whereas the mutation S181G is not expected to disrupt the structure of the protein, this site does score high as a possible phosphorylation site for a number of kinases involved in DNA damage repair, supporting the hypothesis that the cancer cells containing this mutation have lost their ability to respond to transforming DNA damage with programmed cell death. FAF1 antagonizes WNT signaling by promoting *β*-catenin degradation in the proteasome [21], a function that may be lost after the point mutation. The elevated DNA binding activity of the protooncogenic transcription factor E2F1 (see Figure 3) could be caused by its interaction with LEF-1 [20], consecutive to persistent WNT signaling. The RNA level of IGF2BP1 (IMP-1), a stress-responsive regulator of mRNA stability, is highly upregulated in the tumor compared to muscle as well as bone (see Table 2(c)). IGF2BP1 is a transcriptional target of the WNT pathway that regulates NF-*κ*B activity (see Table 2(e)) and is antiapoptotic [22, 23]. IGF2BP1 may be upregulated as a consequence of mutated FAF1 not being able to suppress WNT signaling in this specific cancer. Of note, WNT pathway overactivity may not be required for transformation; rather the persistence of a WNT pathway signal due to the lack of a FAF1-mediated termination signal may suffice.

The role of WNT signaling in sarcoma has been subject to debate (e.g., [24]). In metastatic leiomyosarcoma, *β*-Catenin may accumulate in the nucleus despite a relatively weak expression of WNT [25]. This may be due to WNT signal activation via noncanonical ligands [26] or to *β*-Catenin binding the nuclear receptor NR4A2 and releasing it from the corepressor protein LEF-1 [27]. Of note, in the cancer under study here, the mRNA level of NR4A2 was overexpressed 10-fold compared to bone and 3-fold compared to muscle. The results from this study are consistent with the possibility that a lack of termination in the WNT signal, rather than its overactivation, could contribute to sarcomatous transformation. Such a mechanism may be reflected in an upregulation of downstream targets, even though overexpression of WNT pathway components is not detectable.

Other mutations, beside FAF1, are less likely to be causative for the cancer. EPHA3 was revealed as mutated in this cancer by DNA exome sequencing and RNASeq. EPHA3 is a receptor tyrosine kinase that is frequently mutated in lung cancer. Tumor-suppressive effects of wild-type EPHA3 can be overridden by dominant negative EPHA3 somatic mutations [28]. This mechanism is unlikely to play a role in this sarcoma as the detected mutation is located far N-terminally on the extracellular Ephrin binding domain not on the intracellular kinase domain.

FAF1 has been described to act as a tumor suppressor gene [29]. Its depletion due to chromosome breakage can affect prognosis in glioblastoma patients [30, 31]. Single nucleotide polymorphisms in FAF1 are associated with a risk for gastric cancer [32]. While numerous FAF1 mutations are associated with various cancers, none of these genetic changes in the TCGA database affects the amino acid position 181 (Table 4).

The major upregulations identified in this tumor comprise muscle-specific gene products (transcription factors: CAAC binding; proteomics: transgelin, transgelin-2; RNA: anoctamin-4; and immunohistochemistry: smooth muscle actin) and calcium-regulating molecules (proteomics: calumenin, S100-A11, reticulocalbin-3, and 78 kD glucose-regulated protein). The muscle-specific gene products confirm this recurrent sarcoma as a leiomyosarcoma (the first tumor, distal to the site of the recurring one, had been diagnosed as an osteosarcoma). Calcium is one of the major second messengers in smooth muscle cells. Its uptake is regulated by potential-sensitive ion channels in the cell membrane and by the activities of various receptors. Calcium is stored in the sarcoplasmic reticulum, from where it can be released to facilitate actin-myosin interaction and tension generation. Phosphorylation of the myosin light chain by a calmodulin-regulated enzyme is important for contraction. The upregulation of gene products associated with migration and invasion (osteopontin, MMP1, vimentin, filamin-A, and *β*-actin) and gene products for extracellular matrix molecules and their modulators (fibronectin, collagen, ITGBL1, and MXRA5) reflects the invasive nature of this cancer.

The recurrence of a sarcoma after 14 years has two probable explanations, either it is due to a cancer predisposition syndrome based on a germ-line mutation (the age of the patient weakens this hypothesis) or the second tumor is a metastatic colony of the first that was reactivated after dormancy. The location of the sarcoma in the same extremity and proximal to a preceding mesenchymal cancer (and therefore in its natural path of dissemination) implied the probability that this was a relapse in a metastatic site. The different histologic assessment as osteosarcoma in the first occurrence and leiomyosarcoma as the second cancer does not necessarily negate that. Mixed histology [33, 34] and transdifferentiation [35–37] have been described for sarcomatous tumors. Of note, however, in this scenario osteosarcoma seems to more commonly follow leiomyosarcoma than precede it. Material from the first cancer of this patient was not accessible to us. It is very plausible that this could have been a mineralized leiomyosarcoma. In those tumors, the differential diagnosis from osteosarcoma can be difficult [38]. The extracompartmental location of the first tumor supports this interpretation.

On the molecular pathology level, sarcomas fall into two groups, comprising tumors with simple karyotypes (with pathogenetic translocations or specific genetic mutations) and tumors with very complex karyotypes (overt chromosome and genomic instability with numerous gains and losses) [39]. Some molecular alterations that lead to carcinogenesis can be defined in absolute terms. They include gain-of-function mutations or chromosome translocations that transform protooncogenes to oncogenes. However, other changes are relative to the normal tissue of origin, such as pathway overactivity or overexpression on the protein or RNA levels. We have combined the analysis of absolute changes (DNA exome sequence, RNA sequence) with the analysis of relative changes using skeletal muscle and bone as reference organs (protein-DNA array, 2D gel electrophoresis, and RNA expression levels). This choice was determined in part by tissue availability after surgery and was intended to aid in the distinction of osteosarcoma from myosarcoma. While a more accurate reference point for a leiomyosarcoma would have been smooth muscle, we believe that the comparison to striated muscle is sufficient to allow the assessment of tumor specific changes. We have measured RNA, DNA, and protein with various assays. Similar assessments in the future should also include chromosome analysis for possible translocations.

In the first-line defense against cancer, the gradual replacement of conventional chemotherapy with molecularly targeted agents has opened the possibility to tailor drug treatments to particular tumors. This transition necessitates the characterization of the molecular lesions that are causative for the transformation of healthy cells into cancerous cells, because drugs need to be matched with the underlying carcinogenic defect to be effective. An additional caveat, caused by unique genetic changes in the primary tumor, can affect drug transport and metabolism and needs to be taken into consideration. In this case, the RNA levels for genes that regulate transport and metabolism were extensively skewed in the tumor tissue as compared to muscle and bone (see Table 3), implying potential challenges to chemotherapy. An advanced molecular treatment strategy for cancer will rely on the molecular definition of drug target, drug transport, and drug metabolism pharmacogenetics in the primary tumor. Consecutive to cancer dissemination, it will also require adjustments to account for the genetic changes in the metastases.

The cost of health care has been under increasing scrutiny. The treatment of cancer patients is expensive, in particular when hospitalization is required, and further in cases of end-of-life care. Avoidable expenditures are generated by suboptimal treatment decisions that result in low efficacy or high toxicity of anticancer regimens. Molecular medicine has the potential to preempt those problems and reduce wasteful spending. Yet, it requires the upfront cost of molecular cancer examination. The analysis performed in this study required about $11,000.- in nonsalary expenses to perform. While it has not identified a drug target, it has specified possible confines for drug treatment. The costs for molecular analysis need to be weighed against the societal cost derived from lost productivity in the workforce, disrupted lives of families, and premature deaths. While currently limited drug options constitute the major constraint to the approach taken here, the foreseeable future will bring an increasing spectrum of molecularly targeted drugs along with faster and cheaper technologies for the molecular assessment of cancers. They will facilitate the clinical translation of our approach.

## Figures and Tables

**Table 1:** DNA exome SNPs. The DNA exomes for tumor, muscle, and bone were sequenced. The results were filtered in the following order: (1) different genotype in tumor from muscle and bone, with muscle and bone being identical to each other, (2) delete mutations with low confidence (value of 20 or lower) in all 3 tissues, (3) delete unidentified genes, (4) delete mutations that are homozygous reference in the tumor, (5) delete mutations that have a MAF in dbSNP >10%, (6) delete low impact and modifier mutations. The gene names are part of the key in the left column. In this column, results on bold font represent SNPs that were confirmed on the RNA level by RNASeq. The data files have been submitted to the NCBI short read archive (SRA) under the accession number SRP052797 (biosamples SAMN03316820, SAMN03316821, SAMN03316822).

Key	Chromosome	Position	Reference	Alternate	dbSNP ID	dbSNP MAF	ESP MAF (All)	ESP MAF (EA)	ESP MAF (AA)	Consensus impact	Bone/muscle genotype	Bone overall depth	Bone allele depths	Bone quality	Muscle overall depth	Muscle allele depths	Muscle quality	Tumor genotype	Tumor overall depth	Tumor allele depths	Tumor quality
**17_7479998_T_EIF4A1**	17	7479998	C	T	.	—	—	—	—	Moderate	0/0	175	184,0	99	103	108,0	99	0/1	133	79,68	99
**3_89259200_T_EPHA3**	3	89259200	C	T	.	—	—	—	—	Moderate	0/0	43	45,0	99	117	123,0	99	0/1	72	30,48	99
**1_51204545_C_FAF1**	1	51204545	T	C	.	—	—	—	—	Moderate	0/0	87	91,0	99	108	113,0	99	0/1	85	66,27	99
**12_30834620_A_IPO8**	12	30834620	C	A	.	—	—	—	—	Moderate	0/0	68	71,2	99	120	126,0	99	0/1	80	70,18	99
**11_101834425_A_KIAA1377**	11	101834425	G	A	.	—	—	—	—	Moderate	0/0	36	37,1	90	67	70,0	99	0/1	86	31,63	99
**4_41682102_C_LIMCH1**	4	41682102	G	C	.	—	0.000077	0	0.000227	Moderate	0/0	71	74,0	99	96	101,0	99	0/1	153	120,49	99
**5_36985326_G_NIPBL**	5	36985326	A	G	.	—	—	—	—	Moderate	0/0	6	6,0	18	35	36,0	81	0/1	20	10,12	99
3_77623789_A_ROBO2	3	77623789	AG	A	.	—	—	—	—	High	0/0	22	NA	54	29	NA	87	0/1	37	NA	99
2_217300095_A_SMARCAL1	2	217300095	ATTGCATCAACGTCGTGG	A	.	—	—	—	—	High	0/0	95	NA	99	122	NA	99	0/1	99	NA	99
2_152236045_T_TAA_TNFAIP6	2	152236045	TA	T/TAA	rs35060021	—	—	—	—	High	0/2	19	NA	1	24	NA	57	0/1	17	NA	38
3_52020669_T_ACY1	3	52020669	G	T	.	—	—	—	—	Moderate	0/0	130	136,0	99	86	90,0	99	0/1	80	45,43	99
9_117130734_G_AKNA	9	117130734	C	G	.	—	—	—	—	Moderate	0/0	27	28,0	78	30	31,0	90	0/1	50	30,24	99
12_6030354_A_ANO2	12	6030354	C	A	.	—	—	—	—	Moderate	0/0	101	106,0	99	114	120,0	99	0/1	135	104,45	99
18_10487667_A_APCDD1	18	10487667	G	A	.	—	—	—	—	Moderate	0/0	43	45,0	99	38	40,0	99	0/1	50	38,16	99
7_34118560_C_BMPER	7	34118560	G	C	.	—	—	—	—	Moderate	0/0	69	72,0	99	101	106,0	99	0/1	71	63,14	99
15_24921273_T_C15orf2	15	24921273	G	T	.	—	—	—	—	Moderate	0/0	12	12,0	36	38	39,0	99	0/1	26	22,6	99
19_54483249_C_CACNG8	19	54483249	T	C	.	—	—	—	—	Moderate	0/0	147	154,0	99	103	108,0	99	0/1	152	136,32	99
10_16893265_C_CUBN	10	16893265	G	C	.	—	—	—	—	Moderate	0/0	57	60,0	99	90	94,0	99	0/1	40	34,10	99
7_148489854_C_CUL1	7	148489854	A	C	.	—	—	—	—	Moderate	0/0	73	76,0	99	65	68,0	99	0/1	57	42,21	99
17_41566894_C_DHX8	17	41566894	G	C	.	—	—	—	—	Moderate	0/0	106	111,0	99	88	92,0	99	0/1	124	94,42	99
20_61512358_T_DIDO1	20	61512358	G	T	rs73304513	0.0417	0.045932	0.000838	0.135456	Moderate	0/0	6	0,0	18	2	0,0	6	0/1	4	0,0	21
16_23703526_A_ERN2	16	23703526	G	A	.	—	—	—	—	Moderate	0/0	81	85,0	99	98	103,0	99	0/1	91	60,41	99
3_197880164_G_FAM157A	3	197880164	GCAGCAGCAA	G	.	—	—	—	—	Moderate	0/0	21	NA	25	25	NA	1	0/1	31	NA	6
14_45644287_G_FANCM	14	45644287	A	G	.	0.0009	—	—	—	Moderate	0/0	13	13,0	33	31	32,0	87	0/1	12	10,2	14
1_89637524_C_GBP7	1	89637524	G	C	.	—	—	—	—	Moderate	0/0	171	179,0	99	201	211,0	99	0/1	206	161,67	99
7_42003933_C_GLI3	7	42003933	G	C	.	—	—	—	—	Moderate	0/0	87	91,0	99	81	85,0	99	0/1	85	61,32	99
17_4837170_T_GP1BA	17	4837170	C	T	.	—	—	—	—	Moderate	0/0	16	16,0	45	9	9,0	15	0/1	11	10,1	1
14_24635161_G_IRF9	14	24635161	T	G	.	—	—	—	—	Moderate	0/0	66	69,0	99	43	45,0	99	0/1	36	25,13	99
15_42133096_A_JMJD7-PLA2G4B	15	42133096	G	A	.	—	—	—	—	Moderate	0/0	111	116,0	99	75	78,0	99	0/1	80	74,14	92
17_40271678_A_KAT2A	17	40271678	G	A	.	—	—	—	—	Moderate	0/0	65	68,0	99	58	61,0	99	0/1	56	30,32	99
8_73848894_G_KCNB2	8	73848894	A	G	.	—	—	—	—	Moderate	0/0	15	15,0	33	24	25,0	66	0/1	30	15,17	99
19_50827053_T_KCNC3	19	50827053	C	T	.	—	—	—	—	Moderate	0/0	220	231,0	99	136	143,0	99	0/1	160	130,46	99
17_21319069_A_KCNJ12	17	21319069	G	A	rs76265595	—	—	—	—	Moderate	0/0	23	24,1	63	43	43,4	40	0/1	28	25,5	60
12_53011932_C_KRT73	12	53011932	T	C	.	—	—	—	—	Moderate	0/0	146	153,0	99	157	165,0	99	0/1	159	117,58	99
X_75004584_A_MAGEE2	X	75004584	C	A	.	—	—	—	—	Moderate	0/0	29	30,0	81	48	50,0	99	0/1	38	26,16	99
5_140182157_A_PCDHA3	5	140182157	G	A	.	—	—	—	—	Moderate	0/0	180	189,0	99	153	161,0	99	0/1	150	132,34	99
15_42133096_A_PLA2G4B	15	42133096	G	A	.	—	—	—	—	Moderate	0/0	111	116,0	99	75	78,0	99	0/1	80	74,14	92
10_96005840_C_PLCE1	10	96005840	T	C	.	—	—	—	—	Moderate	0/0	81	85,1	99	73	76,2	99	0/1	87	55,40	99
1_166816805_G_POGK	1	166816805	C	G	.	—	—	—	—	Moderate	0/0	84	88,0	99	82	86,0	99	0/1	63	49,20	99
12_111020739_T_PPTC7	12	111020739	TCGC	T	rs71083132	—	—	—	—	Moderate	0/0	18	NA	30	14	NA	18	0/1	19	NA	10
19_804293_A_PTBP1	19	804293	C	A	.	—	—	—	—	Moderate	0/0	120	126,1	99	71	74,1	99	0/1	75	53,30	99
7_156451175_C_RNF32	7	156451175	G	C	.	—	—	—	—	Moderate	0/0	55	57,0	99	67	70,0	99	0/1	59	42,23	99
3_52020669_T_RP11-155D18.11	3	52020669	G	T	.	—	—	—	—	Moderate	0/0	130	136,0	99	86	90,0	99	0/1	80	45,43	99
21_43838624_T_UBASH3A	21	43838624	G	T	.	—	—	—	—	Moderate	0/0	70	73,0	99	45	47,0	99	0/1	89	76,21	99
19_58773857_A_ZNF544	19	58773857	G	A	.	—	—	—	—	Moderate	0/0	46	48,0	99	43	45,0	99	0/1	71	51,26	99
5_16465723_C_ZNF622	5	16465723	T	C	.	—	—	—	—	Moderate	0/0	17	17,0	39	14	14,0	33	0/1	27	14,15	99

**(a):** 

		log_2_-fold change
		Muscle
Tumor over muscle		
NRG1	Neuregulin 1	9.909
KSR2	Kinase suppressor of ras 2	9.675
MMP13	Matrix metallopeptidase 13 (collagenase 3)	9.528
SPP1	Secreted phosphoprotein 1	9.098
INHBA	Inhibin, beta A	8.570
COL11A1	Collagen, type XI, alpha 1	8.564
MMP9	Matrix metallopeptidase 9 (gelatinase B, 92 kda gelatinase, 92 kda type IV collagenase)	8.552
FBN2	Fibrillin 2	8.495
LRRC15	Leucine rich repeat containing 15	8.429
MMP11	Matrix metallopeptidase 11 (stromelysin 3)	8.336
E2F7	E2F transcription factor 7	8.314
PRAME	Preferentially expressed antigen in melanoma	8.179
PTK7	PTK7 protein tyrosine kinase 7	7.996
DSCAM	Down syndrome cell adhesion molecule	7.761
RGS4	Regulator of G-protein signaling 4	7.633
CNIH3	Cornichon homolog 3 (Drosophila)	7.632
OR10V1	Olfactory receptor, family 10, subfamily V, member 1	7.610
CEP55	Centrosomal protein 55 kda	7.583
WNT5B	Wingless-type MMTV integration site family, member 5B	7.569
CA12	Carbonic anhydrase XII	7.520
GALNT5	UDP-N-acetyl-alpha-D-galactosamine:polypeptide N-acetylgalactosaminyltransferase 5 (GalNAc-T5)	7.517

**(b):** 

		log_2_-fold change
		Bone
Tumor over bone		
ROS1	c-ros oncogene 1, receptor tyrosine kinase	10.987
GREM1	Gremlin 1	10.604
NPTX1	Neuronal pentraxin I	8.813
GJB2	Gap junction protein, beta 2, 26 kDa	8.597
CREB3L1	cAMP responsive element binding protein 3-like 1	8.506
KRT14	Keratin 14	8.351
SERPINE1	Serpin peptidase inhibitor, clade E (nexin, plasminogen activator inhibitor type 1), member 1	8.288
HOXD10	Homeobox D10	8.105
ALPK2	Alpha-Kinase 2	7.783
POU3F2	POU class 3 homeobox 2	7.530
POSTN	Periostin, osteoblast specific factor	7.497
NAA11	N(alpha)-acetyltransferase 11, NatA catalytic subunit	7.439
STC2	Stanniocalcin 2	7.434
WNT5A	Wingless-type MMTV integration site family, member 5A	7.412
IGFN1	Immunoglobulin-like and fibronectin type III domain containing 1	7.387
STC1	Stanniocalcin 1	7.385
FAM180A	Family with sequence similarity 180, member A	7.315
KRT17	Keratin 17	7.305
BBOX1	Butyrobetaine (gamma), 2-oxoglutarate dioxygenase (gamma-butyrobetaine hydroxylase) 1	7.277
MAGEA1	Melanoma antigen family A, 1 (directs expression of antigen MZ2-E)	7.267

**(c):** 

		log_2_-fold change	
		Muscle	Bone
Tumor over bone/muscle			
ANO4	Anoctamin 4 (TMEM16D), transmembrane calcium-activated chloride channel, facilitates smooth muscle contraction	9.937	8.542
SLCO1B3	(OATP1B3) Solute carrier organic anion transporter family, member 1B3	9.569	9.760
MARCH4	Membrane-associated ring finger (C3HC4) 4, E3 ubiquitin ligase, located predominantly to the endoplasmic reticulum	9.538	7.406
IGF2BP1	Insulin-like growth factor 2 mRNA binding protein 1 binds to and stabilizes mRNA	9.440	9.631
ADAMTS16	ADAM metallopeptidase with thrombospondin type 1 motif, 16; zinc-dependent protease	9.260	8.450
SOX11	SRY (sex determining region Y)-box 11, important in brain development	9.178	9.954
HAPLN1	Hyaluronan and proteoglycan link protein 1 stabilizes aggregates of aggrecan and hyaluronan, giving cartilage its tensile strength and elasticity	8.951	8.142
MUC15	Mucin 15, cell surface associated	8.801	7.992
HOXB9	Homeobox B9	8.773	7.378
MAGEC2	Melanoma antigen family C, 2	8.693	8.884
ST6GALNAC5	ST6 (alpha-N-acetyl-neuraminyl-2,3-beta-galactosyl-1,3)-N-acetylgalactosaminide alpha-2,6-sialyltransferase 5	7.848	8.038
C11orf41	Chromosome 11 open reading frame 41	7.781	8.141
MMP1	Matrix metallopeptidase 1 (interstitial collagenase)	7.455	7.646

**(d):** 

Symbol	Name	log_2_-fold change		log_2_-fold change	
		Muscle		Bone	
Tumor over bone/muscle					
FN1	Fibronectin 1	7.328	6.293	6.457	19.860
COL1A1	Collagen, type I, alpha 1	6.768	3.706	5.868	11.934
CCND1	Cyclin D1	5.595	3.958	5.098	9.637
RGS1	Regulator of G-protein signaling 1	5.118	1.056	4.653	2.533
ITGBL1	Integrin, beta-like 1 (with EGF-like repeat domains)	5.071	3.177	3.895	12.415
COL1A2	Collagen, type I, alpha 2	4.852	8.756	4.910	14.503
MXRA5	Matrix-remodelling associated 5	4.834	1.352	4.631	2.685
POSTN	Periostin, osteoblast specific factor	4.513	5.870	7.497	1.267
PLOD2	Procollagen-lysine, 2-oxoglutarate 5-dioxygenase 2	4.285	1.801	4.023	3.730
COL5A2	Collagen, type V, alpha 2	4.275	1.297	4.833	1.517
COL3A1	Collagen, type III, alpha 1	4.003	13.118	5.801	6.500
SEMA3C	Sema domain, immunoglobulin domain (Ig), short basic domain, secreted	3.954	1.164	4.215	1.675
FBN1	Fibrillin 1	3.912	6.957	4.018	11.148
AEBP1	AE binding protein 1	3.808	3.212	4.002	4.843
SIK1	Salt-inducible kinase 1	3.805	1.219	3.566	2.484
SERPINH1	Serpin peptidase inhibitor, clade H (heat shock protein 47), member 1	3.706	1.652	4.145	2.098
ANTXR1	Anthrax toxin receptor 1	3.670	3.789	3.690	6.445

**(e):** 

Symbol	Name	log_2_-fold change		log_2_-fold change	
		Muscle		Bone	
HELLS	Helicase, lymphoid-specific	5.315602	0.155898	−0.82713	20.2489
**TNFRSF11A**	**Tumor necrosis factor receptor superfamily, member 11a, and NFKB activator**	**3.017922**	**0.03091**	**1.400993**	**0.202453**
NFKBID	Nuclear factor of kappa light polypeptide gene enhancer in B-cells inhibitor, delta	2.655352	0	−1.47615	0.504341
**TNFRSF25**	**Tumor necrosis factor receptor superfamily, member 25**	**2.432959**	**0.046388**	**0.163954**	**0.490795**
NFKBIE	Nuclear factor of kappa light polypeptide gene enhancer in B-cells inhibitor, epsilon	2.121015	0.062395	0.311441	0.43382
**TNF**	**Tumor necrosis factor**	**1.847997**	**0**	**−1.42101**	**0.03733**
**TNFRSF10D**	**Tumor necrosis factor receptor superfamily, member 10d, decoy with truncated death domain**	**1.754888**	**0.172968**	**−0.1757**	**1.183343**
**TNFRSF1B**	**Tumor necrosis factor receptor superfamily, member 1B**	**1.473438**	**0.709902**	**−0.97011**	**6.729401**
**TNFRSF10A**	**Tumor necrosis factor receptor superfamily, member 10a**	**1.411898**	**0.828219**	**0.357701**	**3.004386**
NFKBIB	Nuclear factor of kappa light polypeptide gene enhancer in B-cells inhibitor, beta	1.193993	1.209816	0.46055	3.484692
**TNFRSF10B**	**Tumor necrosis factor receptor superfamily, member 10b**	**1.094157**	**1.908597**	**0.425446**	**5.242006**
**TNFRSF21**	**Tumor necrosis factor receptor superfamily, member 21**	**1.055762**	**3.882038**	**0.745815**	**8.305711**
**TNFRSF1A**	**Tumor necrosis factor receptor superfamily, member 1A**	**0.875167**	**3.790938**	**−0.11707**	**13.0344**
NFKBIZ	Nuclear factor of kappa light polypeptide gene enhancer in B-cells inhibitor, zeta	0.575974	3.698951	0.344657	7.493136
**RIPK1**	**Receptor (TNFRSF)-interacting serine-threonine kinase 1**	**0.494467**	**3.11749**	**−1.08854**	**16.1392**
NKRF	NFKB repressing factor	0.475722	2.156087	−0.86397	9.434166
NFKB1	Nuclear factor of kappa light polypeptide gene enhancer in B-cells 1	0.432959	2.054532	−0.65865	7.568586
CHUK	Conserved helix-loop-helix ubiquitous kinase	0.176126	2.961281	−1.20204	13.30333
RELA	V-rel reticuloendotheliosis viral oncogene homolog A (avian)	0.013848	1.263837	0.287167	1.803044
NFKB2	Nuclear factor of kappa light polypeptide gene enhancer in B-cells 2 (p49/p100)	−0.08641	0.312993	0.485882	0.360442
NKAPP1	NFKB activating protein pseudogene 1	−0.10692	0.825177	−0.99449	2.655392
**TNFRSF10C**	**Tumor necrosis factor receptor superfamily, member 10c, decoy without an intracellular domain**	**−0.152**	**0.064676**	**−5.56891**	**6.321515**
NKIRAS2	NFKB inhibitor interacting Ras-like 2	−0.60768	1.095135	−0.88758	2.299946
NFKBIL1	Nuclear factor of kappa light polypeptide gene enhancer in B-cells inhibitor-like 1	−0.8719	0.141933	−0.08357	0.140418
NKIRAS1	NFKB inhibitor interacting Ras-like 1	−0.87428	6.296399	1.467735	2.122983
TANK	TRAF family member-associated NFKB activator	−0.983	6.777124	−1.51908	16.95872
NKAP	NFKB activating protein	−1.35735	14.22458	−0.78243	16.46287
NFKBIA	Nuclear factor of kappa light polypeptide gene enhancer in B-cells inhibitor, alpha	−1.85483	40.32743	−0.31802	23.95275
NKAPL	NFKB activating protein-like	−5.57347	5.875743	−1.40215	0.534643

**Table:** (a) Transport

Gene ID	Symbol	Name	Tumor bone	Tumor muscle
			log_2_-fold change	log_2_-fold change
650655	ABCA17P	ATP-binding cassette, subfamily A (ABC1), member 17, pseudogene	−1.360	−*2.116 *
24	ABCA4	ATP-binding cassette, subfamily A (ABC1), member 4	−*2.038 *	−*2.802 *

6555	SLC10A2	Solute carrier family 10 (sodium/bile acid cotransporter family), member 2	−1.962	**−2.112**
345274	SLC10A6	Solute carrier family 10 (sodium/bile acid cotransporter family), member 6	−*2.623 *	−*3.240 *
6563	SLC14A1	Solute carrier family 14 (urea transporter), member 1 (Kidd blood group)	**−3.074**	**−3.152**
6565	SLC15A2	Solute carrier family 15 (H+/peptide transporter), member 2	**−2.027**	**−2.152**
6566	SLC16A1	Solute carrier family 16, member 1 (monocarboxylic acid transporter 1)	−1.401	−*2.170 *
117247	SLC16A10	Solute carrier family 16, member 10 (aromatic amino acid transporter)	−*2.113 *	−*2.848 *
6567	SLC16A2	Solute carrier family 16, member 2 (monocarboxylic acid transporter 8)	−*3.242 *	−*3.848 *
10786	SLC17A3	Solute carrier family 17 (sodium phosphate), member 3	**−2.868**	**−2.959**
6571	SLC18A2	Solute carrier family 18 (vesicular monoamine), member 2	**−2.087**	**−2.194**
6573	SLC19A1	Solute carrier family 19 (folate transporter), member 1	**−2.099**	**−2.203**
10560	SLC19A2	Solute carrier family 19 (thiamine transporter), member 2	−1.820	−*2.548 *
80704	SLC19A3	Solute carrier family 19, member 3	**−3.331**	**−3.478**
387775	SLC22A10	Solute carrier family 22, member 10	−*5.711 *	−*6.085 *
9390	SLC22A13	Solute carrier family 22 (organic anion transporter), member 13	−*2.623 *	−*3.217 *
85413	SLC22A16	Solute carrier family 22 (organic cation/carnitine transporter), member 16	**−8.101**	**−7.299**
51310	SLC22A17	Solute carrier family 22, member 17	−1.475	−*2.175 *
5002	SLC22A18	Solute carrier family 22, member 18	−*2.038 *	−*2.722 *
6582	SLC22A2	Solute carrier family 22 (organic cation transporter), member 2	−*4.793 *	−*5.216 *
6581	SLC22A3	Solute carrier family 22 (extraneuronal monoamine transporter), member 3	−*2.554 *	−*3.182 *
6583	SLC22A4	Solute carrier family 22 (organic cation/ergothioneine transporter), member 4	**−3.603**	**−3.776**
151295	SLC23A3	Solute carrier family 23 (nucleobase transporters), member 3	−*2.109 *	−*2.848 *
10478	SLC25A17	Solute carrier family 25 (mitochondrial carrier), member 17	−1.311	−*2.077 *
83733	SLC25A18	Solute carrier family 25 (mitochondrial carrier), member 18	−1.846	−*2.570 *
788	SLC25A20	Solute carrier family 25 (carnitine/acylcarnitine translocase), member 20	**−2.105**	**−2.207**
89874	SLC25A21	Solute carrier family 25 (mitochondrial oxodicarboxylate carrier), member 21	**−2.962**	**−3.105**
51312	SLC25A37	Solute carrier family 25, member 37	**−4.633**	**−4.866**
51629	SLC25A39	Solute carrier family 25, member 39	**−2.585**	**−2.737**
203427	SLC25A43	Solute carrier family 25, member 43	−1.325	−*2.088 *
65012	SLC26A10	Solute carrier family 26, member 10	−*3.896 *	−*4.472 *
115111	SLC26A7	Solute carrier family 26, member 7	−*3.431 *	−*4.018 *
116369	SLC26A8	Solute carrier family 26, member 8	**−5.354**	**−5.536**
115019	SLC26A9	Solute carrier family 26, member 9	−1.623	−*2.419 *
11001	SLC27A2	Solute carrier family 27 (fatty acid transporter), member 2	**−4.278**	**−4.487**
64078	SLC28A3	Solute carrier family 28 (sodium-coupled nucleoside transporter), member 3	**−4.543**	**−4.812**
222962	SLC29A4	Solute carrier family 29 (nucleoside transporters), member 4	−1.962	**−2.045**
81031	SLC2A10	Solute carrier family 2 (facilitated glucose transporter), member 10	−*2.532 *	−*3.170 *
6518	SLC2A5	Solute carrier family 2 (facilitated glucose/fructose transporter), member 5	**−3.647**	**−3.821**
55532	SLC30A10	Solute carrier family 30, member 10	**−3.547**	**−3.725**
7782	SLC30A4	Solute carrier family 30 (zinc transporter), member 4	−*2.832 *	−*3.372 *
6569	SLC34A1	Solute carrier family 34 (sodium phosphate), member 1	**−4.716**	**−4.941**
340146	SLC35D3	Solute carrier family 35, member D3	**−5.662**	**−5.906**
54733	SLC35F2	Solute carrier family 35, member F2	−1.433	−*2.170 *
206358	SLC36A1	Solute carrier family 36 (proton/amino acid symporter), member 1	**−2.265**	**−2.345**
285641	SLC36A3	Solute carrier family 36 (proton/amino acid symporter), member 3	**−2.284**	**−2.393**
54020	SLC37A1	Solute carrier family 37 (glycerol-3-phosphate transporter), member 1	**−2.112**	**−2.212**
2542	SLC37A4	Solute carrier family 37 (glucose-6-phosphate transporter), member 4	**−2.117**	**−2.219**
151258	SLC38A11	Solute carrier family 38, member 11	−*2.410 *	−*3.085 *
55089	SLC38A4	Solute carrier family 38, member 4	−1.837	−*2.548 *
92745	SLC38A5	Solute carrier family 38, member 5	−1.994	**−2.152**
91252	SLC39A13	Solute carrier family 39 (zinc transporter), member 13	−1.301	−*2.070 *
23516	SLC39A14	Solute carrier family 39 (zinc transporter), member 14	−*2.569 *	−*3.188 *
29985	SLC39A3	Solute carrier family 39 (zinc transporter), member 3	**−2.032**	**−2.152**
283375	SLC39A5	Solute carrier family 39 (metal ion transporter), member 5	−1.623	−*2.370 *
7922	SLC39A7	Solute carrier family 39 (zinc transporter), member 7	−1.273	−*2.058 *
30061	SLC40A1	Solute carrier family 40 (iron-regulated transporter), member 1	**−3.612**	**−3.796**
84102	SLC41A2	Solute carrier family 41, member 2	−1.293	−*2.070 *
8501	SLC43A1	Solute carrier family 43, member 1	**−2.013**	**−2.152**
57153	SLC44A2	Solute carrier family 44, member 2	**−2.047**	**−2.152**
50651	SLC45A1	Solute carrier family 45, member 1	−*2.038 *	−*2.722 *
146802	SLC47A2	Solute carrier family 47, member 2	−1.962	**−2.026**
6521	SLC4A1	Solute carrier family 4, anion exchanger, member 1	**−6.513**	**−6.453**
57282	SLC4A10	Solute carrier family 4, sodium bicarbonate transporter, member 10	**−2.640**	**−2.753**
83959	SLC4A11	Solute carrier family 4, sodium borate transporter, member 11	−1.846	−*2.569 *
6508	SLC4A3	Solute carrier family 4, anion exchanger, member 3	−1.261	−*2.045 *
8671	SLC4A4	Solute carrier family 4, sodium bicarbonate cotransporter, member 4	−*2.445 *	−*3.113 *
9497	SLC4A7	Solute carrier family 4, sodium bicarbonate cotransporter, member 7	−*2.717 *	−*3.307 *
6523	SLC5A1	Solute carrier family 5 (sodium/glucose cotransporter), member 1	**−2.547**	**−2.705**
159963	SLC5A12	Solute carrier family 5 (sodium/glucose cotransporter), member 12	−*4.753 *	−*5.206 *
6527	SLC5A4	Solute carrier family 5 (low affinity glucose cotransporter), member 4	**−3.969**	**−4.249**
6540	SLC6A13	Solute carrier family 6 (neurotransmitter transporter, GABA), member 13	−1.328	−*2.092 *
55117	SLC6A15	Solute carrier family 6 (neutral amino acid transporter), member 15	−1.623	−*2.333 *
388662	SLC6A17	Solute carrier family 6, member 17	−*2.038 *	−*2.728 *
54716	SLC6A20	Solute carrier family 6 (proline IMINO transporter), member 20	−1.697	−*2.433 *
6532	SLC6A4	Solute carrier family 6 (neurotransmitter transporter, serotonin), member 4	**−2.512**	**−2.611**
6534	SLC6A7	Solute carrier family 6 (neurotransmitter transporter, L-proline), member 7	−*2.623 *	−*3.228 *
56301	SLC7A10	Solute carrier family 7, (neutral amino acid transporter, y+ system) member 10	**−3.421**	**−3.603**
6547	SLC8A3	Solute carrier family 8 (sodium/calcium exchanger), member 3	**−2.204**	**−2.309**
285335	SLC9A10	Solute carrier family 9, member 10	−1.208	−*2.000 *
6549	SLC9A2	Solute carrier family 9 (sodium/hydrogen exchanger), member 2	−*2.038 *	−*2.706 *
9368	SLC9A3R1	Solute carrier family 9 (sodium/hydrogen exchanger), member 3 regulator 1	**−2.760**	**−2.846**
9351	SLC9A3R2	Solute carrier family 9 (sodium/hydrogen exchanger), member 3 regulator 2	−1.889	−*2.619 *
84679	SLC9A7	Solute carrier family 9 (sodium/hydrogen exchanger), member 7	−*3.347 *	−*3.935 *
10599	SLCO1B1	Solute carrier organic anion transporter family, member 1B1	−*3.360 *	−*3.977 *
28234	SLCO1B3	Solute carrier organic anion transporter family, member 1B3	−*9.760 *	−*9.569 *
6578	SLCO2A1	Solute carrier organic anion transporter family, member 2A1	−*2.432 *	−*3.096 *
28232	SLCO3A1	Solute carrier organic anion transporter family, member 3A1	**−2.032**	**−2.152**
353189	SLCO4C1	Solute carrier organic anion transporter family, member 4C1	**−6.850**	**−6.611**

**Table:** (b) Metabolism

Gene ID	Symbol	Name	Tumor bone	Tumor muscle
			log_2_-fold change	log_2_-fold Cchange
1583	CYP11A1	Cytochrome P450, family 11, subfamily A, polypeptide 1	−1.623	−*2.396 *
1589	CYP21A2	Cytochrome P450, family 21, subfamily A, polypeptide 2	−1.962	**−2.036**
1591	CYP24A1	Cytochrome P450, family 24, subfamily A, polypeptide 1	−*5.208 *	−*5.577 *
1592	CYP26A1	Cytochrome P450, family 26, subfamily A, polypeptide 1	−*2.623 *	−*3.257 *
1594	CYP27B1	Cytochrome P450, family 27, subfamily B, polypeptide 1	−1.846	−*2.585 *
339761	CYP27C1	Cytochrome P450, family 27, subfamily C, polypeptide 1	−*3.585 *	−*4.178 *
1553	CYP2A13	Cytochrome P450, family 2, subfamily A, polypeptide 13	−1.962	**−2.035**
1580	CYP4B1	Cytochrome P450, family 4, subfamily B, polypeptide 1	**−4.820**	**−5.065**
66002	CYP4F12	Cytochrome P450, family 4, subfamily F, polypeptide 12	**−6.070**	**−6.195**
8529	CYP4F2	Cytochrome P450, family 4, subfamily F, polypeptide 2	**−7.769**	**−7.060**
4051	CYP4F3	Cytochrome P450, family 4, subfamily F, polypeptide 3	**−8.244**	**−7.491**
11283	CYP4F8	Cytochrome P450, family 4, subfamily F, polypeptide 8	**−5.006**	**−5.270**
260293	CYP4X1	Cytochrome P450, family 4, subfamily X, polypeptide 1	**−2.476**	**−2.580**
9420	CYP7B1	Cytochrome P450, family 7, subfamily B, polypeptide 1	−*2.502 *	−*3.170 *

2326	FMO1	Flavin containing monooxygenase 1	−*4.360 *	−*4.859 *
2327	FMO2	Flavin containing monooxygenase 2 (nonfunctional)	**−4.074**	**−4.337**
2328	FMO3	Flavin containing monooxygenase 3	**−4.036**	**−4.309**
388714	FMO6P	Flavin containing monooxygenase 6 pseudogene	**−2.569**	**−2.737**

493869	GPX8	Glutathione peroxidase 8 (putative)	−*2.814 *	−*3.371 *
2938	GSTA1	Glutathione S-transferase alpha 1	−1.623	−*2.363 *
2939	GSTA2	Glutathione S-transferase alpha 2	−*2.038 *	−*2.741 *
2941	GSTA4	Glutathione S-transferase alpha 4	−1.492	−*2.188 *
2953	GSTT2	Glutathione S-transferase theta 2	−*4.682 *	−*5.170 *
653689	GSTT2B	Glutathione S-transferase theta 2B (gene/pseudogene)	−*3.739 *	−*4.307 *

84779	NAA11	N(alpha)-acetyltransferase 11, NatA catalytic subunit	−*7.439 *	−*7.996 *
9027	NAT8	N-acetyltransferase 8 (GCN5-related, putative)	**−2.769**	**−2.920**

7358	UGDH	UDP-glucose 6-dehydrogenase	−*2.333 *	−*3.018 *
55757	UGGT2	UDP-glucose glycoprotein glucosyltransferase 2	−1.604	−*2.271 *
10720	UGT2B11	UDP glucuronosyltransferase 2 family, polypeptide B11	**−3.586**	**−3.768**
7367	UGT2B17	UDP glucuronosyltransferase 2 family, polypeptide B17	**−2.836**	**−2.959**
54490	UGT2B28	UDP glucuronosyltransferase 2 family, polypeptide B28	**−3.132**	**−3.284**
167127	UGT3A2	UDP glycosyltransferase 3 family, polypeptide A2	**−4.716**	**−4.907**

**Table 4:** FAF1 mutations in various cancers. FAF1 mutations listed in the TCGA data base were identified without restriction to any type of cancer. For the location of the affected domains on the protein compare Figure 2.

AA	Mutation	Cancer	Domain
N4S	Missense	Cutaneous melanoma	
I10S	Missense	Stomach adenocarcinoma	UBA
E21^*^	Nonsense	Cutaneous melanoma	UBA
E25K	Missense	Uterine endometrioid carcinoma	UBA
V38_splice	Splice	Stomach adenocarcinoma	UBA
P86fs	FS del	Colorectal adenocarcinoma	
G123_splice	Splice	Uterine endometrioid carcinoma	UB1
P136H	Missense	Colorectal adenocarcinoma	UB1
T147M	Missense	Brain lower grade glioma	UB1
D149Y	Missense	Uterine endometrioid carcinoma	UB1
L159V	Missense	Lung adenocarcinoma	UB1
K163N	Missense	Uterine endometrioid carcinoma	UB1
L170F	Missense	Cutaneous melanoma	
G184_splice	Splice	Colorectal cancer	
Q187H	Missense	Stomach adenocarcinoma	
S214N	Missense	Colorectal adenocarcinoma	UB2
R222I	Missense	Uterine endometrioid carcinoma	UB2
E238D	Missense	Lung adenocarcinoma	UB2
P241S	Missense	Uterine endometrioid carcinoma	UB2
T245A	Missense	Renal clear cell carcinoma	UB2
M249V	Missense	Uterine endometrioid carcinoma	UB2
E280K	Missense	Brain lower grade glioma	
G293^*^	Nonsense	Colorectal cancer	
T300I	Missense	Colorectal adenocarcinoma	
D305H	Missense	Lung adenocarcinoma	
E308Q	Missense	Lung adenocarcinoma	
A316V	Missense	Stomach adenocarcinoma	
K319fs	FS ins	Head and neck squamous cell carcinoma	
R344G	Missense	Stomach adenocarcinoma	UAS
F353I	Missense	Cutaneous melanoma	UAS
L379V	Missense	Breast invasive carcinoma	UAS
C396F	Missense	Cutaneous melanoma	UAS
S459^*^	Nonsense	Uterine endometrioid carcinoma	UAS
G469_splice	Splice	Glioblastoma multiforme	UAS
R509G	Missense	Lung squamous cell carcinoma	
E510fs	FS del	Cutaneous melanoma	
R516C	Missense	Uterine endometrioid carcinoma	
A534V	Missense	Stomach adenocarcinoma	
F537L	Missense	Stomach adenocarcinoma	
E551^*^	Nonsense	Lung adenocarcinoma	
R554W	Missense	Colorectal cancer	
S582I	Missense	Lung adenocarcinoma	UBX
F585L	Missense	Uterine endometrioid carcinoma	UBX
E587^*^	Nonsense	Lung adenocarcinoma	UBX
A592V	Missense	Stomach adenocarcinoma	UBX
W610^*^	Nonsense	Breast invasive carcinoma	UBX
D611Y	Missense	Lung adenocarcinoma	UBX
E635fs	FS del	Brain lower grade glioma	UBX
P640fs	FS del	Cutaneous melanoma	UBX
